# MicroRNAs Regulate Metabolic Phenotypes During Multicellular Tumor Spheroids Progression

**DOI:** 10.3389/fonc.2020.582396

**Published:** 2020-12-04

**Authors:** Erick Andrés Muciño-Olmos, Aarón Vázquez-Jiménez, Diana Elena López-Esparza, Vilma Maldonado, Mahara Valverde, Osbaldo Resendis-Antonio

**Affiliations:** ^1^ Human Systems Biology Lab, National Institute of Genomic Medicine, Mexico City, Mexico; ^2^ PhD Program in Biomedical Sciences, UNAM, Mexico City, Mexico; ^3^ Unidad de Investigación en Reproducción Humana, Instituto Nacional de Perinatología “Isidro Espinosa de los Reyes”—Facultad de Química, Universidad Nacional Autónoma de México, Ciudad de México, Mexico; ^4^ Epigenetic Laboratory, Instituto Nacional de Medicina Genómica, Ciudad de México, Mexico; ^5^ Department of Genomic Medicine and Environmental Toxicology, Institute for Biomedical Research, National Autonomous University of Mexico, Mexico City, Mexico; ^6^ Coordinación de La Investigación Científica -Red de Apoyo a La Investigación, UNAM, Mexico City, Mexico

**Keywords:** cancer metabolism, miRNA-mRNA interaction, multicellular tumor spheroids, bioinformatics, miRNA target prediction

## Abstract

During tumor progression, cancer cells rewire their metabolism to face their bioenergetic demands. In recent years, microRNAs (miRNAs) have emerged as regulatory elements that inhibit the translation and stability of crucial mRNAs, some of them causing direct metabolic alterations in cancer. In this study, we investigated the relationship between miRNAs and their targets mRNAs that control metabolism, and how this fine-tuned regulation is diversified depending on the tumor stage. To do so, we implemented a paired analysis of RNA-seq and small RNA-seq in a breast cancer cell line (MCF7). The cell line was cultured in multicellular tumor spheroid (MCTS) and monoculture conditions. For MCTS, we selected two-time points during their development to recapitulate a proliferative and quiescent stage and contrast their miRNA and mRNA expression patterns associated with metabolism. As a result, we identified a set of new direct putative regulatory interactions between miRNAs and metabolic mRNAs representative for proliferative and quiescent stages. Notably, our study allows us to suggest that miR-3143 regulates the carbon metabolism by targeting hexokinase-2. Also, we found that the overexpression of several miRNAs could directly overturn the expression of mRNAs that control glycerophospholipid and N-Glycan metabolism. While this set of miRNAs downregulates their expression in the quiescent stage, the same set is upregulated in proliferative stages. This last finding suggests an additional metabolic switch of the above mentioned metabolic pathways between the quiescent and proliferative stages. Our results contribute to a better understanding of how miRNAs modulate the metabolic landscape in breast cancer MCTS, which eventually will help to design new strategies to mitigate cancer phenotype.

## Introduction

MicroRNAs (miRNAs) are small non-coding RNAs of approximately 22 nucleotides in size that are related to critical regulatory functions in a plethora of biological processes, associated with healthy and dysfunctional physiological states (1, 2). The most common and well-documented functions of miRNAs are to be endogenous negative regulators of the translation and contribute to the mRNAs instability. This regulatory capacity is carried out through direct base pairing to the target sites in the 3′ untranslated regions of an mRNA (3). To date, there are more than 1,900 mature human miRNAs according to the latest build (build 22) of the Sanger Centre miRNA database miRBase (http://www.mirbase.org). To highlight their regulatory relevance, last estimations suggest that this set of miRNAs target at least 60% of human mRNAs (4). For this reason, miRNAs have surged as crucial post-transcriptional regulators whose dysregulation can be tightly associated with aberrant gene expression in complex human diseases such as cancer (5).

In cancer research, there are a myriad of publications uncovering the role of miRNAs during their pathogenesis and progression (6, 7). Despite their broad dysregulation, miRNAs can be functionally classified as oncogenes or tumor suppressors (8). This fact leads to most of the human tumors present a unique pattern of expression, setting a large and complex network of interactions between oncogenes being activated and the loss of various tumor suppressors. Regardless of this variation in the genetic profiles, these interactions between oncogenes and tumor suppressors can directly or indirectly induce metabolic alterations that favor the survival and growth of the tumoral cells (9). For instance, there is experimental evidence that miRNA-143 down-regulates Hexokinase 2 (*HK2)* which promotes cancer progression and the reduction in glucose metabolism (10). Hence, miRNAs participate in the control of metabolic reprogramming by regulating the expression of mRNAs whose protein products regulate directly metabolic machinery (11, 12). Despite the substantial progress in this area, we still need to complete the puzzle and establish the role of miRNAs on metabolic rewiring into cancer progression and how this could be exploited as therapeutic targets in cancer.

A major impediment in cancer treatment is the drug resistance due to the heterogeneous subpopulations of cells within the tumor with different cell-cycle phases (13, 14). The cell cycle is the mechanism associated with proliferation, cellular division and DNA replication. It is mainly divided into four phases: G1-phase where proteins necessary for S-phase progression are accumulated, the S-phase period where DNA synthesis occurs, the G2-phase where proteins required for mitosis are produced and the M-phase phase where mitosis and separation occurs (15). Also, cells may occasionally exit from the cell-cycle and enter a phase of quiescence called the G0-phase (16). With this in mind, models that closely resemble human cancer cell-cycle heterogeneity are essential for understanding the growth mechanisms and for the development of new treatments. For this reason, MultiCellular Tumor Spheroids (MCTS) models have been used to study and reproduce the gradients between proliferating and quiescent cells, cell-cell interaction, low drug penetration and resistance of quiescent cells located in the deepest and hypoxic regions (17–19).

In the present study, we proceeded with a experimental and computational pipeline to report new plausible miRNA-mRNA pairs interactions involved in the metabolic rewiring of cancer, using an *in vitro* MCTS model of a luminal A breast cancer cell line (MCF7), the most prevalent subtype in women worldwide (20). To explore the regulatory effect of miRNAs over metabolism, we accomplished a longitudinal study of the MCTS in a period of 19 days of progression. In this interval of time, the MCTS owns cell populations with different cell cycle stages, particularly proliferative and quiescent cells, which can provide differences in the metabolic phenotype as time passes. Using this model, we conducted a whole transcriptome analysis, including RNAs and small-RNAs, to describe how miRNAs influence metabolism. Our paired analysis of RNAseq and small RNAseq allowed us to conclude that whereas glycerophospholipid and glucose metabolism are down-regulated in proliferative enriched MCTS, oxidative phosphorylation, and amino acid biosynthesis is down-regulated in quiescent enriched MCTS. Overall our study highlighted the crucial role of miRNAs to guide the metabolic phenotype in MCF7 MCTS. Notably, the set of miRNAs modulating the expression of various metabolic mRNAs seems to be a promising avenue to design new *in vitro* strategies to mitigate the malignant phenotype in cancer.

## Materials and Methods

### Experimental Procedures

#### MCF7 Monoculture

Breast cancer cell line MCF7 (ATCC HTB-22TM, Manassa, VA, USA) was grown in DMEM (ATCC 30-2002, Manassa, VA, USA) containing 4 mM L-glutamine, 4,500 mg/L glucose, 1 mM sodium pyruvate, and 1,500 mg/L sodium. Media was supplemented with 10% v/v of FBS (ATCC 30-2020, Manassa, VA, USA). The media was replaced every 2 days with fresh media. Cells were incubated under a humidified atmosphere with 5% CO_2_ and 95% air at 37°C. For all experiments, 70–80% confluent monoculture with less than 9 passages were used. MCF7 cell line was validated using STR analysis.

#### Generation and Disaggregation of Multicellular Tumor Spheroids (MCTS) Cultures from MCF7

The generation of MCTS was carried out using a liquid overlay technique. A single-cell suspension of MCF7 at a density of 1x10^6^ cells was loaded into 12.5 cm^2^ suspension culture flasks (UltraCruz sc-200257, Tex, USA) containing 5 mL of L-15 media (ATCC 30-2008, Manassa, VA, USA) supplemented with 5% v/v of FBS. Flasks were placed in an orbital incubator at 37°C under constant orbital shaking of 59 rpm for 6 and 19 days (21). The media was replaced every 2 days with fresh media.

For disaggregation, the 6 and 19 day-old MCTS were harvested and transferred to 1.5 mL tubes. The MCTS were washed with PBS 1X (VWR 97062-732, PA, USA). Accutase (Invitrogen 00-4555-56, CA, USA) was added and the reaction was carried out for 45 min. at 37°C with orbital shaking. Every 5 min. the MCTS were mixed gently by pipetting during the accutase reaction. To ensure optimal disaggregation, Trypsin-EDTA (0.25% Trypsin, 1mM EDTA) solution was added for 5 min at 37°C. The trypsin reaction was stopped by adding media with FBS in a 1:1 ratio. Finally, cells were collected by centrifugation and were suspended in 0.1% BSA in PBS (Cell Signaling Technology BSA #9998, USA) solution.

#### MCTS Diameter Distribution

**Figure 1:**
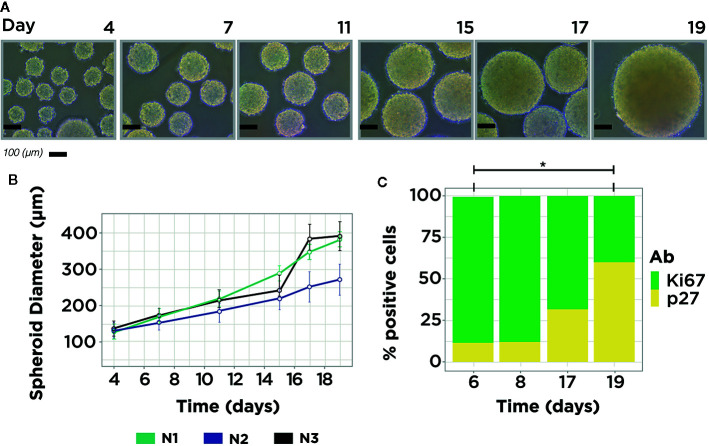
MCF7 MCTS Model. **(A)** Representative images from MCF7 MCTS culture showing size differences along time. **(B)** MCTS diameter distribution measurements, average MCTS measure per time point = 67, n = 3. **(C)** Immunophenotyping of MCTS subpopulations with flow cytometry using Ki67 and p27 markers in four temporal conditions: day 6, 8, 17, and 19. The expression of both markers showed statistical difference in the comparison between days 6 and 19. Single asterisk indicates a statistical difference, P <0.05 (unpaired t-test with equality of variances in normalized measurements, t = ± 4.4629; df = 4).

Diameter distribution of MCTS at 4, 7, 11, 15, 17, and 19 day-old time was estimated by two steps. First, we took photos directly to the MCTS culture, ensuring that almost all MCTS are positioned at the center of the flask. Photos were taken using a Nikon Eclipse TS100 Inverted Microscope. Following this, the images were analyzed through the MorphLibJ package to directly estimate the Feret diameter of all the MCTS in each picture (22). The Feret diameter value reported in **Figure 1** represents the mean value of all MCTS properly measured for each time point. The diameter distribution was calculated for three independent MCTS cultures using the time points mentioned above (**Table S1**).

#### MCTS Culture Time Points Selection

To evaluate the abundance of cells in proliferative and quiescent stages inside the MCTS through time, we selected four-time points: 6, 8, 17, and 19 days. The first two time points (6 and 8 days) of MCTS culture were chosen because after 6 days almost every cell has been aggregated to form an MCTS. The next two time points were picked because after 20 days of MCTS culture necrotic population started to appear, a condition that was avoided in our study.

#### Immunophenotyping

The 6-, 8-, 17-, and 19-day old MCTS cultures were disaggregated as described in *Generation and Disaggregation of Multicellular Tumor Spheroids (MCTS) Cultures from MCF7* section. Therefore, we took an aliquot of 3x10^6^ cells of each MCTS culture. Fixation was carried out using PFA 4% for 10 min. at room temperature and rinsing three times with PBS 1X. Subsequently, the permeabilization was accomplished by adding ice-cold methanol (90%) during 30 min. in ice. Previous to the antibody staining, the methanol was removed by washing with PBS 1X and 1x10^6^ cells were aliquoted. Then, the cells were rinsed with 3 mL of incubation buffer (0.1% BSA in PBS) three times. Staining was made by resuspending the cells with the Ki67-Alexa Fluor^®^ 488 Conjugate (CST 11882, Massachusetts, USA) and p27-PE Conjugate (CST 12184, Massachusetts, USA) primary antibodies (1:50) and incubating for 1 h at room temperature. Before cell cytometry analysis, cells were resuspended in 500 µL of PBS 1X. Finally, the immunophenotyping was done in a FACSAria Cell Sorter.

#### RNA Extraction Method for MCTS and Monoculture

Total RNA was isolated from MCF7 monoculture, 6- and 19-day old MCTS by a TRIzol (Invitrogen, CA, USA) adapted protocol. Particular adjustments previous to the TRIzol extraction were made for both culture conditions. In the monoculture, we used a 70–80% confluent culture, then we poured 1 mL of TRIzol directly to the culture dish and homogenized until all cells were detached from the dish. This step was assisted by the use of a scraper. In the 6- and 19-day old MCTS culture, the MCTS were retrieved by centrifugation and resuspended in 1 mL of TRIzol. The solution was homogenized vigorously until there were no cellular lumps. The subsequent steps described below were used for both culture conditions. We added 10 µL of triton 2% (Merck T8787, St. Louis, USA) and incubated for 10 min. at room temperature. Next, we added 200 µL of chloroform, mixed through inversion and incubated for 5 min. at room temperature. Tubes were centrifuged at 12,000 rpm during 15 min. at 18°C. The aqueous phase was retrieved, we added 0.25 µL of glycogen as co-precipitation reagent (ThermoFisher R0561, Waltham, USA) and isopropanol was added in a 1:1 ratio (regarding TRIzol). The mixture was homogenized gently, incubated for 20 min. at −20°C and centrifuged at 13,000 rpm during 45 min. at 18°C. Finally, the pellet was washed three times with ethanol 75%, dissolved in ultrapure DDW (not treated with DEPC) and stored at −80°C.

#### RNA-Seq and Small RNA-Seq Library Construction and Sequencing

Previous to the library construccion we ensured that all RNA samples have a minimum amount of 1 µg and the integrity value of the samples have a RIN >7. The NGS was performed on total RNA isolated as described in the previous section using the NextSeq platform (Illumina, Inc.). The library construction for RNA-Seq and small RNA-Seq was made following the TruSeq RNA Library Prep Kit (Illumina, Inc.) and the CD Small RNA Library Prep Kit (Illumina, Inc.), respectively. The datasets presented in this study can be found in online repositories. The names of the repository and accession number can be found below: ArrayExpress, Accession: E-MTAB-9741 (https://www.ebi.ac.uk/arrayexpress/experiments/E-MTAB-9741/)

### Bioinformatic Analysis

#### Quality Determination and Pre-Processing

All the FASTQ files undergo quality evaluation using FastQC software version 3 (http://www.bioinformatics.babraham.ac.uk/projects/fastqc/). The resulting output files were summarized using the MultiQC tool (23). Only RNA-Seq FASTQ files were pre-processed to remove low-quality information in the 3′ and 5′ end using Trimmomatic version 0.36 (24).

#### Alignment and Entity Quantification

For RNA-Seq FASTQ pre-processed files, the alignment was made using Kallisto version 0.43.1 (25). The results were summarized using the tximport package in R (26). For small RNA-Seq the alignment was made using Bowtie2 version 2.3.2 (27) with the parameters suggested in (28) and the human genome GRCh38 construction. The resulting SAM files were summarized using FeatureCounts (29) with the hsa.gff file from miRBase v22.

#### Exploratory Analysis and Differential Expression Analysis

The processed data was inspected for normalized data through principal component analysis (PCA) plot (**Figure S1**). The differential expression analysis was carried out with edgeR package (30) version 3.26.8 for RNA-Seq and small RNA-Seq reads.

#### miRNAs Target Prediction for Metabolic mRNAs

To only obtain the Differential Expressed (DE) metabolic mRNAs in each condition, we filtered the DE mRNAs using the KEGG metabolic pathways gene list using an FDR <0.05 threshold. Next, we used DE miRNAs and the metabolic DE mRNAs mentioned before to obtain the plausible miRNAs-mRNA pairs calculated with miRGate (31). From miRGate analyses we only used the predictions from Targetscan algorithm and the experimental validated miRNA-mRNA pairs for further filters. We filtered the plausible pairs when the log2FC has an inverse value between miRNAs and the metabolic mRNAs. Finally, the selected pairs were filtered to those who appear at least two times in a metabolic pathway.

#### Pathway Enrichment Analysis

Pathway enrichment analysis was done using two different methods within Webgestalt (32). First method consists of taking the full mRNA list into an over-representation analysis (ORA). Second, gene set enrichment analysis (GSEA) was made using the mRNA differential expressed with a FDR <0.05. We used the KEGG, GO, Panther, and wikipathway datasets.

## Results

### MCTS Culture as a Model of Proliferation and Quiescence

To explore the possible interaction between miRNAs and mRNAs of metabolic genes in cancer, we carried out a MCTS culture with the breast cancer cell line MCF7. We mainly used the human luminal A (ER+, PR+, HER2-) cell line due to its high incidence in women breast cancer patients worldwide (20). In this MCTS model, MCF7 cell suspension was loaded on non-adherent plates to stimulate cell-cell adhesion and promote well-rounded spherical structures. This experimental model, and its implemented protocols, facilitated the tracking of the progression of the MCTS and ensured the production of large batches of MCTS on diverse time points (**Figure 1A**). The Feret Diameter reported in **Figure 1B**. is the average value of MCTS measurements in each time point (**Table S1**). To select the optimal time point where the MCTS is mainly enriched in a cell cycle phase, we assess the presence of a standard marker for proliferation (Ki67) and quiescence (p27) in four-time points using immunophenotyping. Also, to contrast the differences across a classical culture method, we included a 2D model (monoculture) along all the study to compare against a proliferative enriched MCTS and quiescent enriched MCTS. We applied a t-test analysis over the relative composition of the proliferative and quiescent population in monoculture and MCTS to identify two sample times to proceed with NGS data analysis. The statistical analysis showed significant differences in some samples between the expression of Ki67 and p27 (**Figure S2**). As a result of this study, we found that only the comparison between MCTS at 6- and 19-day old showed statistical differences in both cell cycle markers (**Figure 1C**). Consequently, these time points were selected to carry on the transcriptome and small-RNA profiling. These results indicate that MCTS 6-day old are primarily enriched in proliferative cells (P-MCTS), and MCTS 19-day old are principally enriched in quiescent cells (Q-MCTS).

### Bioinformatics Analysis of RNA-Seq and Small RNA-Seq

**Figure 2:**
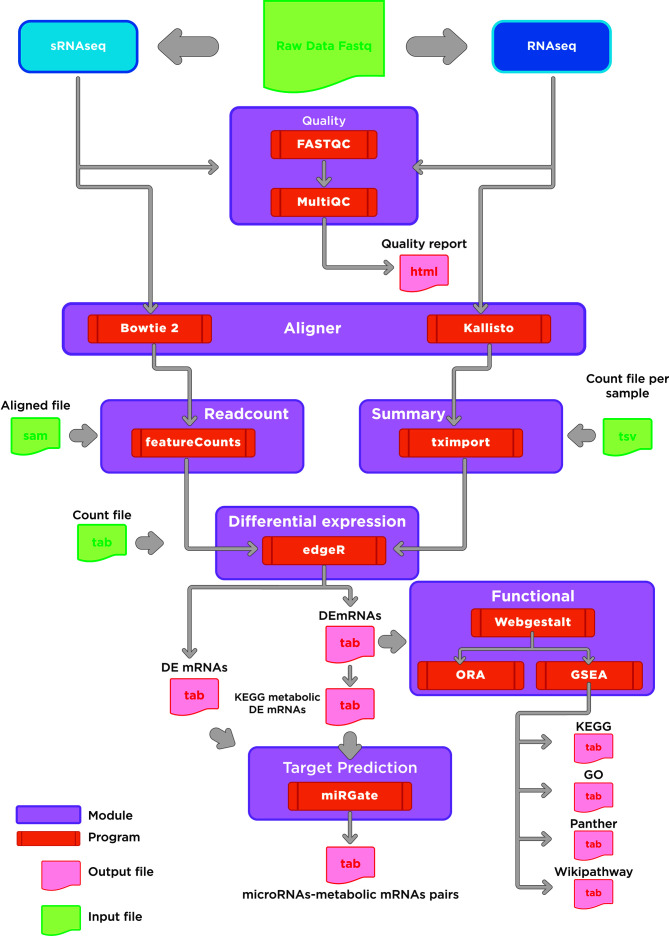
Bioinformatic pipeline workflow. Overview of the general modules implemented for the analysis of mRNA and microRNAs expression. Main modules are indicated in purple. Output files are indicated in light pink. Our workflow shows the software applied in the major steps of RNAseq and small RNA sequencing analysis: 1) Quality check, 2) Alignment, 3) Read count and differential expression, 4) Functional analysis; and 5) miRNAs target prediction.

To date, few computational tools can simultaneously accomplish the analysis of RNA-Seq and small RNA-Seq. Among these algorithms, miARma-Seq provides a multiprocess analysis tool that can undergo these restraints and allow the study of expression profiles of miRNAs and their targeted mRNAs (33). A study combining mRNA and miRNA sequencing can undoubtedly supply a proper framework to assess pair-wise connections between miRNAs and metabolic targets mRNAs. With this aim in mind, we implemented some changes in the original miARma-Seq pipeline, using the best-suited tools for each processing module. The two significant changes in the pipeline were performed in the alignment tool for RNA-Seq data and the enrichment tool. Thus, we set Kallisto and Webgestalt as the new processing tools (**Figure 2**). An in-deep description of the adjustments made for each processing module is described in the methods section. In brief, our pipeline is integrated by a set of modules which embraces classical steps for each technology (mRNA and microRNA) such as quality check, alignment, differential expression, target prediction, and functional analysis. A global view of the entire pipeline implemented in this paper is depicted in **Figure 2**.

#### Differential Expression Analysis and Metabolic mRNAs Target Prediction

**Figure 3:**
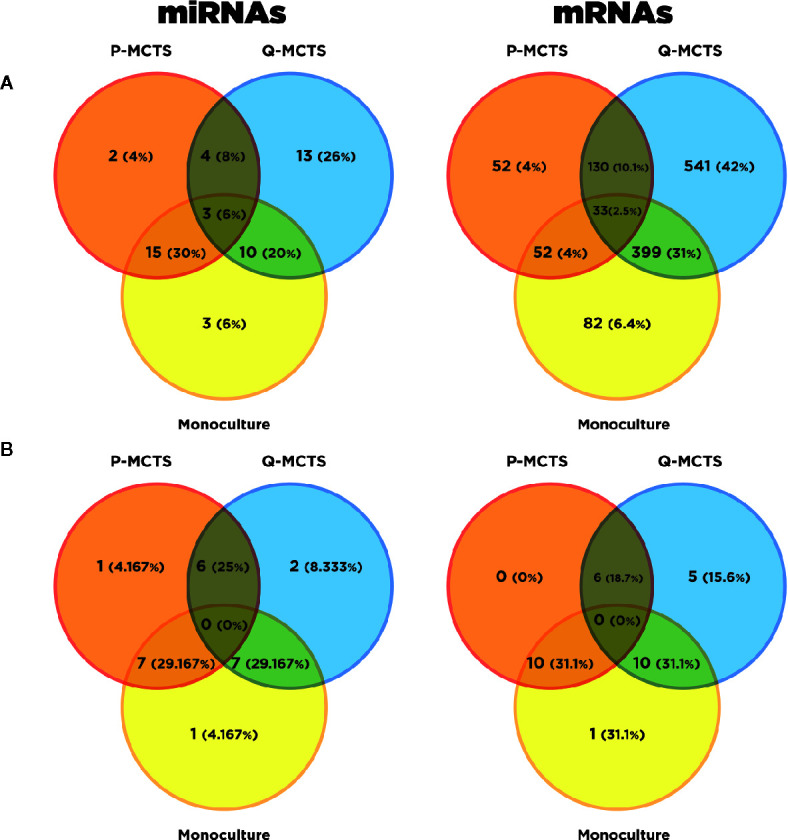
Differential Expression Analysis Overview. **(A)** Venn diagram resulting from the DE mRNAs and DE miRNAs in the conditions comparisons with a specified threshold. Orange, blue, and yellow represent the DE mRNAs in P-MCTS, Q-MCTS and monoculture conditions respectively. **(B)** Venn diagram resulting from the DE metabolic mRNAs subset and DE miRNAs after the target prediction with paired information using miRGate.

In this section, we pursue two aims: explore how miRNA and mRNAs expression changes among conditions; and identify potential miRNAs-mRNA regulatory mechanisms at a metabolic level. To assess the changes in RNA expression profiles among the samples, we obtained the Differential Expressed (DE) profile of mRNAs and miRNAs for each pair-wise comparison among P-MCTS, Q-MCTS, and monoculture condition. From a statistical point of view, we used an FDR <0.05 as a criteria to select those mRNAs and miRNAs differentially expressed between conditions. As a result, we identified a total of 1,289 mRNAs and 35 miRNAs differentially expressed among all pairwise conditions. Particularly, the comparison between P-MCTS and monoculture showed no significant results. Therefore, to get insight of the regulation differences we used only for this comparison a p-value <0.05. By doing so, 15 unique miRNAs were added to the analysis. **Figure 3A** summarize the set of DE mRNAs and miRNAs obtained per comparison with the considerations indicated above.

In order to disentangle the metabolic rewiring accomplished by miRNAs, we carried out a filter within the DE mRNAs to just obtain the subset of mRNAs that codify for metabolic machinery. This last analysis was accomplished by using the KEGG metabolic pathways gene set (34). Subsequently, miRGate was used to characterize *in silico* potential regulatory interactions between the DE metabolic mRNAs and the miRNAs. As a result of this bioinformatic analysis, 32 mRNAs and 24 miRNAs were selected as miRNA-RNAs interactions obtained with the considerations described in the miRNAs target prediction for metabolic mRNAs section. To dissect which of these miRNAs-mRNAs regulatory interactions between miRNAs and metabolic mRNAs are unique or shared across the cultured conditions, we visually recap their comparison in **Figure 3B**. As shown in this last figure, the numerical distribution and percentage of miRNA and their predicted DE mRNA targets shared and specific for each condition are shown in **Figure 3B**. In the next section, we present an in-deep analysis over these pairs of miRNAs-mRNA interactions and its possible functional consequences.

#### Metabolic mRNAs-miRNAs Pairs Functions

In the previous section, we identified a set of 24 miRNAs and 32 mRNAs that could have a regulatory effect in the metabolic pathways for the three comparisons. Based on this set of 24 miRNAs and 32 mRNAs, in this section we explored their pattern of expression and their possible influence on signaling and metabolic pathways for each pairwise condition, see **Tables 1** **–** **3**. In general terms, miRNA-mRNA pairs could influence different signaling and metabolic pathways, obtained as described in *miRNAs Target Prediction for Metabolic mRNAs* section. For instance, in the comparison between Q-MCTS and P-MCTS, we observed an upregulation of mRNAs in fatty acids and lipid metabolism. Also, we noted a downregulation of the biosynthesis of amino acids. Furthermore, mRNAs participating in glycolysis, oxidative phosphorylation, and glycan metabolism do not show a particular preference for a comparison due to the disparity in the pathway usage (**Table 1**). Comparative analysis between Q-MCTS and monoculture showed an up-regulation in mRNAs participating in lipid metabolism, particularly the glycerophospholipid metabolism. Notably, although we had a different set of DE mRNAs in each condition, we observed that mRNAs in glycerophospholipid metabolism remain up-regulated in Q-MCTS with respect to all comparisons.

**Table 1:** Pathways affected by the mRNA-miRNA pairs for the comparison between Q-MCTS and P-MCTS.

		hsa-miR-7974	hsa-miR-181a-5p	hsa-miR-663a	hsa-miR-1184	hsa-miR-3648	hsa-miR-454-3p	hsa-miR-3143	hsa-miR-671-5p	hsa-miR-15a-5p
Glycerophospholipid metabolism	**DGKG**	+/-								
	**PLA2G3**	+/-								
	**PLA2G4C**		+/-							
Ether lipid metabolism	**PLA2G3**	+/-								
	**PLA2G4C**		+/-							
Arachidonic acid metabolism	**PLA2G3**	+/-								
	**PLA2G4C**		+/-							
Linoleic acid metabolism	**PLA2G3**	+/-								
	**PLA2G4C**		+/-							
Alpha-Linolenic acid metabolism	**PLA2G3**	+/-								
	**PLA2G4C**		+/-							
Ras signaling pathway	**PLA2G3**	+/-								
	**PLA2G4C**		+/-							
Phospholipase D signaling pathway	**DGKG**	+/-								
	**PLA2G4C**		+/-							
Choline metabolism in cancer	**DGKG**	+/-								
	**PLA2G4C**		+/-							
Phosphatidylinositol signaling system	**DGKG**	+/-								
	**ITPKB**			-/+	-/+	-/+				
N-Glycan biosynthesis	**MGAT5**				-/+					
	**STT3B**						+/-	+/-	+/-	
Oxidative phosphorylation	**ATP6V1C1**									+/-
	**NDUFB6**				-/+					
Glycolysis/Gluconeogenesis	**HK2**							+/-		
	**PGAM1**				-/+					
Biosynthesis of amino acids	**GLUL**			-/+						
	**PGAM1**				-/+					

To classify the profiles of expression between mRNA target and miRNA, we defined a two-component notation, which indicates their relative expression. Red shows the cases where mRNA overexpress and miRNA downregulate their expressions in Q-MCTS with respect to P-MCTS (+/-). Green means the inverse situation and it was denoted as (-/+).

Among the exclusive overexpressed pathways in monoculture, we stand out the inositol phosphate metabolism, purine metabolism, glycosaminoglycan metabolism, and pathways such as calcium and sphingolipid signaling pathways. The carbon metabolism for this comparison also showed differences in pathway usage (**Table 2**). Finally, the comparison between P-MCTS and monoculture showed a preference for the biosynthesis and degradation of the amino acids, the inositol metabolism, and glycerophospholipid metabolism in P-MCTS. The monoculture condition does not show a particular pathway usage for this comparison; however there is an overexpression in *GFPT2*, suggesting a UDP sugar metabolism preference (**Table 3**).

**Table 2:** Pathways affected by the mRNA-miRNA pairs for the comparison between Q-MCTS and monoculture.

		hsa-miR-7974	hsa-miR-3929	hsa-miR-663a	hsa-miR-3648	hsa-miR-663b	hsa-miR-501-5p	hsa-miR-193a-5p	hsa-miR-362-5p	hsa-miR-3652	hsa-miR-1226-3p
Phosphatidylinositol signaling system	**DGKG**	+/-									
	**INPP5A**		-/+								
	**ITPKB**		-/+	-/+	-/+						
	**PLCB3**					-/+					
Glycerophospholipid metabolism	**DGKG**		+/-								
	**PLA2G3**		+/-								
	**PLD3**							+/-			
Inositol phosphate metabolism	**INPP5A**		-/+								
	**ITPKB**		-/+	-/+	-/+						
	**PLCB3**					-/+					
Purine metabolism	**AMPD2**					-/+					
	**HPRT1**							-/+			
	**ADA**				-/+						
Fructose and mannose metabolism	**HK2**								+/-		
	**KHK**									-/+	
	**GMPPB**										+/-
Glycosaminoglycan biosynthesis - heparan sulfate/heparin	**XYLT2**									-/+	
	**NDST1**									-/+	
Phospholipase D signaling pathway	**DGKG**	+/-									
	**PLCB3**					-/+					
Ether lipid metabolism	**PLA2G3**	+/-									
	**PLD3**						+/-				
Amino sugar and nucleotide sugar metabolism	**HK2**								+/-		
	**GMPPB**										+/-
Carbohydrate digestion and absorption	**HK2**								+/-		
	**PLCB3**					-/+					
Calcium signaling pathway	**ITPKB**		-/+	-/+	-/+						
	**PLCB3**					-/+					
Sphingolipid signaling pathway	**PLCB3**					-/+					
	**CERS6**		-/+								

In the figure, we defined a two-component notation to classify the profiles of expression between mRNA target and miRNA, which indicates their relative expression. Red indicates the cases where mRNA overexpress and miRNA downregulate their expressions in Q-MCTS with respect to monoculture (+/-). Green indicates the inverse situation and it was denoted as (-/+).

**Table 3:** Pathways affected by the mRNA-miRNA pairs for the comparison between P-MCTS and monoculture.

		hsa-miR-429	hsa-miR-454-3p	hsa-miR-940	hsa-miR-320c	hsa-miR-19a-3p	hsa-miR-1226-3p	hsa-miR-492	hsa-miR-4721	hsa-miR-26a-5p	hsa-miR-940
Glycerolipid metabolism	**DGAT2**		+/-								
	**LPIN1**	+/-									
Inositol phosphate metabolism	**PLCB4**		+/-								
	**INPP5J**			+/-							
Phosphatidylinositol signaling system	**PLCB4**		+/-								
	**INPP5J**			+/-							
Valine, leucine and isoleucine degradation	**HMGCS1**				+/-						
	**ACADSB**		+/-			+/-	+/-				
Amino sugar and nucleotide sugar metabolism	**GFPT2**							-/+			
	**GMPPB**			+/-			+/-		+/-		
Glycerophospholipid metabolism	**LPIN1**	+/-									
	**PCYT1A**									+/-	
Biosynthesis of amino acids	**PRPS1**				+/-						
	**PYCR1**										+/-

To classify the profiles of expression between mRNA target and miRNA, we defined a two-component notation, which indicates their relative expression. Red shows the cases where mRNA overexpress and miRNA downregulate their expressions in P-MCTS with respect to monoculture (+/-). Green means the inverse situation, and it was denoted as (-/+).

#### Functional Analysis

**Figure 4:**
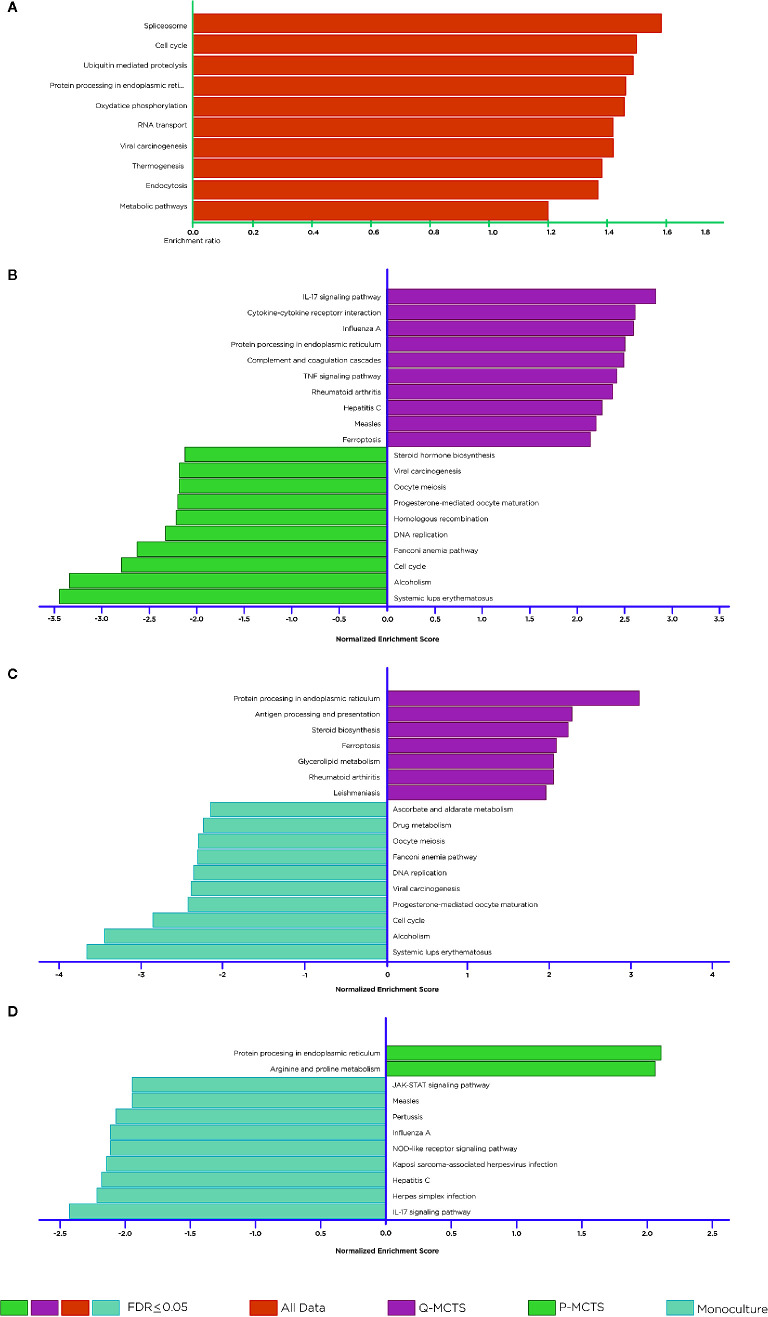
Pathway Enrichment Analysis. Statistical evaluation of the fraction of mRNAs in a particular pathway found in the DE mRNAs across comparisons and in the whole dataset. **(A)** Over-representation analysis. **(B)** GSEA. Comparison between Q-MCTS and P-MCTS. **(C)** GSEA. Comparison between Q-MCTS, and monoculture. **(D)** GSEA. Comparison between P-MCTS and monoculture. Orange, magenta, green and light blue bars show statistically enriched pathways for all data, Q-MCTS, P-MCTS, and monoculture, respectively.

To obtain a global perspective in the pathways usage in the whole mRNA data, we accomplished a gene set enrichment analysis over all the samples together. To this end, we applied an Over Representation Analysis (ORA) and selected those pathways with an FDR ≤0.05. This primary study allowed us to identify pathways that change over all the samples. Interestingly the enrichment suggested pathways involved in the spliceosome, cell cycle, proteolysis, protein processing, RNA transport, carcinogenesis, thermogenesis, endocytosis, and metabolic pathways (**Figure 4A**). The results recapitulate pathways according to the selection of the samples, such as the cell cycle. However, this method did not allow us to distinguish in which condition the pathway was over-represented. Thus, we use a complementary approach to achieve a more specific insight into the pathways usage between comparisons. We carried out a GSEA using different datasets for all pairwise comparisons. **Figures 4B–D** depicts some of the main enrichment pathways defined in the KEGG database. In general terms, we noted that most overrepresented pathways lead to abnormal immune responses, metabolic rewiring, cell division, subversion of cellular signaling pathways, and DNA replication and repair. When comparing Q-MCTS vs. P-MCTS, we observed an up-regulation of mRNAs in inflammatory responses and immune suppression and a downregulation of steroid biosynthesis, cell division, and DNA replication and repair (**Figure 4B**).

Comparative analysis between Q-MCTS and monoculture showed an up-regulation in mRNAs participating in steroid biosynthesis, glycerolipid metabolism and pathways involved in abnormal immune responses, and a downregulation of ascorbate, aldarate and drug metabolism, cell division, and DNA replication and repair (**Figure 4C**). Taking these results together, we noticed that the enriched pathways for the proliferative-like culture methods (P-MCTS and monoculture) are very similar when compared to Q-MCTS. Besides, Q-MCTS maintains the enrichment in pathways involved in abnormal immune responses. Finally, the comparison between P-MCTS and monoculture showed an up-regulation in mRNAs participating in arginine and proline metabolism and a downregulation in the immune responses and the cellular signaling pathways (**Figure 4D**).

## Discussion

This study investigated the possible regulatory interactions between miRNAs and its metabolic mRNAs targets in the human breast cancer cell line MCF7. This aim was particularly carried out by applying and analyzing simultaneously RNA-seq and small RNA-Seq of MCTS and monoculture. As a result, two main findings can be highlighted. First, we provide new insights into the regulatory mechanism by which miRNAs modulate metabolic mRNAs to sustain cancer MCTS. Second, we have accomplished an in-depth bioinformatics analysis to characterize these miRNA regulations, and evaluate their consequences over pathways sustaining phenotype in Q-MCTS, P-MCTS, and monoculture conditions. In the following, we discuss and draw some conclusions for each pairwise culture comparison.

### Q-MCTS vs. P-MCTS Comparison

**Figure 5:**
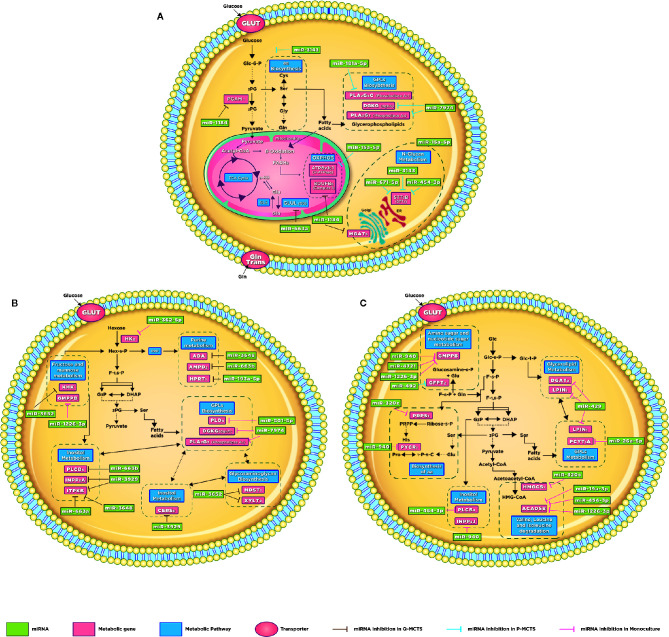
Schematic illustration of altered metabolic pathways. Global metabolic prospect with the miRNAs involvement. **(A)** Q-MCTS and P-MCTS comparison. **(B)** Q-MCTS and Monoculture comparison. **(C)** P-MCTS and Monoculture comparison. The block arrows indicated in brown, blue, and pink show the inhibition in the metabolic pathway for Q-MCT, P-MCTS, and Monoculture, respectively.

The comparison between Q-MCTS and P-MCTS reveals that Q-MCTS primarily have low expression in miRNAs that control the lipid metabolism and hexokinase 2 (HK2), setting a high preference for glycerophospholipid and glycolytic metabolism usage. On the other hand, P-MCTS down-regulates some miRNAs that control the biosynthesis of amino acids and oxidative phosphorylation through the targeting of *NDUFB6* mRNA (**Table 1**). These results agreed with previous observations dealing with miRNAs and cancer cell metabolism. For instance, HK2 is the first rate-limiting enzyme of glycolysis, and its activity has been predicted to be regulated by multiple miRNAs including the confirmed negative regulation by miR-143 in breast cancer cell lines (12). Here, we suggested a new regulatory interaction in HK2 mRNA mainly carried out by miR-3143 and preferently activated in P-MCTS when compared with Q-MCTS (**Figure 5A**).

In addition to the carbon metabolism, differences in the amino acid and lipid metabolism have been reported before and consistently observed in this study, see **Figure 5A** Lipids are essential biosynthesis molecules for organelles and cells. The disturbance in lipid metabolism guided by miRNA regulation is a particular feature of cancer metabolism (35–37). Here, we propose that miR-7974 and miR-181a-5p regulate a set of mRNAs implicated in the lipid metabolism, predominantly the glycerophospholipid metabolism. These miRNAs are downregulated in Q-MCTS suggesting that the lipid metabolism remains active. Meanwhile the lipid metabolism is downregulated in P-MCTS by the list of miRNAs mentioned in **Table 1**. Moreover, cancer cells have an increased demand for amino acids to meet their rapid biosynthesis of proteins, nucleotides and lipids, redox homeostasis, and energy metabolism. There is evidence that miRNAs regulate amino acid catabolism in kidney cancer (38). In our study, we observed that the low expression of miRNAs regulating *GLUL* and *PGAM1* mRNAs in P-MCTS assists their rapid proliferation, maintaining a high expression in these mRNAs to conserve the serine and glutamine pools. In Q-MCTS, the over-expression of miR-663a and miR-1184 can downregulate the amino acid metabolism to mainly depend only on a glycolytic and lipid metabolism (**Figure 5A**).

Regarding the oxidative phosphorylation findings, the overexpression of *ATP6V1C1* in Q-MCTS, while in P-MCTS this mRNA is downregulated by miR-15a-5p. The *ATP6V1C1* gene encodes a component of vacuolar ATPase (V-ATPase). The V-ATPase complex is located at the plasma membrane and plays an important role in tumor growth and metastasis by the increment in H+ secretion, granting tumor cells to survive in hypoxic conditions and the consequent acidic tumor microenvironment (39). Also, in a mouse breast cancer model was shown that the *Atp6v1c1* knockdown reduced invasion and migration (40). Based on these facts, we suggest that the rewiring of the metabolic program in Q-MCTS is due to the hypoxic conditions within the MCTS that aid in acquiring a metastatic phenotype in the quiescent subpopulation, unlike of its proliferative counterpart (**Figure 5A**).

Finally, we found that the dependence in the N-Glycan metabolism is guided by two mRNAs: *STT3B* and *MGAT5*. The expression of these mRNAs show an inverse regulatory phenotype. While *STT3B* mRNA is mainly down-regulated in P-MCTS by a group of miRNAs (miR-3143, miR-671-5p, and miR-454-3p), *MGAT5* mRNA is down-regulated in Q-MCTS by miR-1184. The branching of the N-Glycans is implicated in the regulation of surface levels of glycoproteins such as the epidermal growth factor (EGF) and transforming growth factor-ß (TGF-β) receptors. Also, the ablation of *MGAT5* mRNA in tumor cells leads to less metastatic and less responsive to cytokines phenotype (41), and *STT3B* participates in the epithelial-mesenchymal transition (EMT) in cancer cells (42). For these reasons, we suggested that in Q-MCTS and P-MCTS the metastatic potential could exist, no matter the metabolic profile (**Figure 5A**).

### Q-MCTS vs Monoculture Comparison

The comparison between Q-MCTS and monoculture reveal that Q-MCTS primarily has low expression in some miRNAs that control the glycerophospholipid biosynthesis and the glycolytic metabolism, suggesting a high preference for these metabolic pathways. On the other hand, monoculture down-regulates miRNAs regulating purine metabolism, inositol metabolism, sphingolipid metabolism, and glycosaminoglycan biosynthesis (**Table 2**). The obtained results showed that the down-regulation in the glycolytic metabolism, mainly in HK2 is in agreement with previous cancer metabolism reports. Here, we suggested a new regulation carried out by miR-362-5p on HK2, which is intensified in monoculture and diminished in Q-MCTS (**Figure 5B**). Intriguingly, the down-regulation of HK2, is a frequent feature for the proliferative-like models (P-MCTS and Monoculture), although it is guided by different miRNAs depending on the culture method. These results supply additional evidence pointing out that the Q-MCTS depends on a glycolytic metabolism.

Additionally, the results showed an overexpression of mRNAs of the purine metabolism in the monoculture. We also suggested that this pathway is controlled by miR-3648, miR-663b, and miR193a-5p targeting *ADA*, *AMPD2*, *and HPRT1*, respectively in Q-MCTS (**Figure 5B**). The purines are essential components for RNA and DNA production and provide the cofactors and energy to support cell survival and proliferation (43). In concordance with our findings, the pathway usage correlates with the high proliferative rate seen by immunophenotyping with Ki67 (**Figure S2**), suggesting its essential role in monoculture.

On the other hand, fructose and mannose metabolism seems to have significant changes between these conditions. Based on our analysis, we concluded that these pathways can be altered by two mRNAs: *KHK* and *GMPPB*. The expression of these mRNAs shows an inverse phenotype: while KHK upregulates its expression, GMPPB downregulates its expression. This contrary behavior leads to an unclear pathway usage. In Q-MCTS, we found that *KHK* mRNA is mainly down-regulated by miR-3652, and *GMPPB* mRNA is down-regulated in monoculture by miR-1226-3p. This pathway could fuel the pentose phosphate flux and protein synthesis, indirectly increasing tumor growth (44). Also, there is experimental evidence indicating that fructose can be used by breast cancer cells specifically in glucose-deficiency environments (45) and the upregulation of *KHK* correlates with tumor malignancy and progression (46). Moreover, *GMPPB* overexpression is associated with a favorable prognostic value in endometrial cancer (47). Overall, we concluded that individual participation of these mRNAs may lead to a more severe phenotype for the monoculture model.

As discussed earlier, we observed a frequent disturbance in lipid metabolism through all comparisons. This metabolic preference remains in the comparison between Q-MCTS vs monoculture. Specifically, the glycerophospholipid metabolism is overexpressed in Q-MCTS. Based on our bioinformatics analysis, a possible explanation can be given due to the regulation on two miRNAs. We suggest that miR-501-5p down-regulates *PLD3* and miR-7974 down-regulates *DGKG* and *PLA2G3* in monoculture. Furthermore, *CERS6* participating in the sphingolipid metabolism is overexpressed in monoculture, and is down-regulated by miR-3929 in Q-MCTS, see **Figure 5B**. Together these results showed that there is no particular usage for the lipid metabolism in general. However, the lipid categories can be exclusive to a cell cycle phase, for instance, the glycerophospholipid metabolism being constantly overexpressed in Q-MCTS.

Finally, the down-regulation of Glycosaminoglycan metabolism in Q-MCTS is accomplished through the regulation of miR-3652 over *NDST1* and *XYLT2*. Conversely, in monoculture, these two mRNAs are overexpressed. Another result indicates that the inositol metabolism is up-regulated in monoculture, whereas in Q-MCTS this pathway is down-regulated by miR-663b, miR-3929, miR-3648, and miR-663a. Remarkably, both metabolic pathways are used to fuel signaling processes. In fact, glycosaminoglycans are part of the extracellular matrix (ECM), which conducted interactions with growth factors and cytokines implicated in cancer growth and progression, mainly signaling cascades responsible for regulating angiogenesis, invasion, and metastasis (48). Also, the mRNAs involved in the inositol metabolism can sustain the PI3K-dependent signaling pathways, promoting tumor growth and invasiveness (49). The up-regulation in monoculture for both metabolic pathways suggests that this preference is followed by the high proliferation rates observed, especially in this culture model.

### P-MCTS vs Monoculture Comparison

The comparison between P-MCTS and monoculture reveals that P-MCTS primarily has low expression in miRNAs that control the biosynthesis of amino acids, inositol, valine, leucine, isoleucine, and lipid metabolism. For instance, we found that the monoculture solely down-regulates miR-492, which in turn regulates *GFPT2*, a mRNA participating in amino and nucleotide sugar metabolism (**Table 3**). The phenotype in this metabolic pathway is mainly influenced by two mRNAs: *GFPT2* and *GMPPB*. The expression of these mRNAs shows an inverse phenotype. While one upregulates, the other downregulates its expression. In P-MCTS, *GFPT2* mRNA is mainly down-regulated by miR-492, and *GMPPB* mRNA is down-regulated in monoculture by miR-940, miR4721, and miR-1226-3p. The abundance of the nucleotide pools limits the cancer proliferative capacity (50), suggesting that both models require this pathway to maintain their proliferative capacity, regardless of the pathway regulation.

Additionally, the comparative analysis showed an overexpression of mRNAs involved in the lipid metabolism in the P-MCTS. Mainly, the glycerolipid and glycerophospholipid metabolism are a consistent result across all comparisons, showing a preference in the MCTS models (Q-MCTS and P-MCTS). However, in monoculture this pathway is down-regulated by miR-429 targeting *DGAT2*, *LPIN1*, and miR-26a-5p targeting *PCYT1A* (**Figure 5C**). These results correlated with the fact that the highest levels in lipid profiles are found in the most aggressive breast tumors (51), suggesting that the lipid metabolism can promote malignancy in the MCTS models, despite the differences in the proliferation rates.

Likewise, our analysis showed an overexpression in the biosynthesis and degradation of amino acid in P-MCTS. Additionally, in monoculture, we suggested that the amino acid biosynthesis is down-regulated by miR-320c and miR-940 targeting *PRPS1* and PYCR1, respectively. Also, the amino acid degradation is down-regulated by miR-320c targeting *HMGCS1*, and *ACADSB* is down-regulated by a set of miRNAs; miR-19a-3p, miR454-3p, and miR-1226-3p (**Figure 5C**). These results agree with a proteome study in 3D cancer cultures (52), which suggest that spheroid cultures rely on amino acid utilization.

Finally, the overexpression in the inositol metabolism is accompanied by the up-regulation of *PLCB4* and *INPP5J* in P-MCTS. Conversely, in monoculture, this pathway is down-regulated by miR-454-3p and miR-940, targeting these two mRNAs. These results suggest that this pathway and their corresponding fueled signaling pathways, such as PI3K, may be upregulated in the MCTS model.

### Altered Signaling Pathways

Our analysis mainly focused on metabolic alterations; however, other pathways presented significant perturbations to explore more deeply. For instance, we found that pathways associated with cell division, DNA replication and repair are enriched in the proliferative-like models (P-MCTS and Monoculture). As we expected, these results are in agreement with the demands for sustaining a rapid proliferation. Complementary, inflammatory responses and immune suppression are enriched pathways for the Q-MCTS (**Figure 4**). Furthermore, our analysis makes evidence that Q-MCTS phenotype points to the overrepresentation in inflammatory cytokines, which have a key role in cancer progression *via* the stimulation of the epithelial-to-mesenchymal transition, and augmentation of metastasis in cancer (53). Another important and recurrent result is the overrepresentation of the ferroptosis. This pathway is characterized by iron-dependent accumulation of reactive oxygen species (ROS) within the cell, leading to cell death. Therefore, activation of ferroptosis derives in a selective elimination of some tumor cells (54). Together, these results suggest that the Q-MCTS can selectively enhance an invasive phenotype. As a whole, these observations mirror an orchestrated response to integrate the cancer phenotype, and it is in agreement with the fact that different tumor clonal subpopulations can diversify their task to maintain their growth and malignancy (14).

### Outlook

Metabolic reprogramming plays an essential role in tumor development and metastasis. Besides, targeting cancer metabolism remains a great promise in developing anti-cancer therapies. As we propose here, several miRNAs may be controlling various metabolic pathways during MCTS progression. Although the model used in this study has limitations and the suggested interactions require subsequent experimental validation, we postulate the presence of new miRNAs-mRNA interactions that can modulate the metabolic landscape in the different cell populations that coexist during MCTS growth. In this context, other reports showed that miRNAs deregulation does not alter the growth in normal cells, but it has a reduction in the growth of cancer cells (55–57). Although the progress in the effectiveness of miRNAs/anti-miRs delivery *in vivo* is still a major obstacle in clinics, our results highlight the miRNAs associated with metabolic changes in breast cancer as possible markers in peripheral blood since they are less labile than mRNAs and as therapeutic targets in cancer. Likewise, many functional activities of miRNAs and targets are unknown during tumor progression, so it is important to study them to understand how tumor cells propitiate its phenotype. Hopefully, our approaches and findings can be useful to verify experimentally these microRNA-mRNA interactions and explore their pragmatic implications for developing more effective cancer treatments that will target metabolic alteration within tumor subpopulations.

## Data Availability Statement

The datasets presented in this study can be found in online repositories. The names of the repository/repositories and accession number(s) can be found below: ArrayExpress, Accession: E-MTAB-9741 (https://www.ebi.ac.uk/arrayexpress/experiments/E-MTAB-9741/).

## Author Contributions

EM-O and OR-A designed the project and developed the methods. EM-O performed the experiments and the bioinformatic analysis. EM-O and AV-J developed the functional analysis. EM-O, AV-J, OR-A, VM, MV, and DL-E contributed with discussions in all sections. All authors contributed to the article and approved the submitted version.

## Funding

The authors thank the financial support from an internal grant of the National Institute of Genomic Medicine.

## Conflict of Interest

The authors declare that the research was conducted in the absence of any commercial or financial relationships that could be construed as a potential conflict of interest.
