# Supplementary material for: MicroRNAs Regulate Metabolic Phenotypes During Multicellular Tumor Spheroids Progression
Source: Front Oncol. 2020 Dec 4;10:582396. doi: 10.3389/fonc.2020.582396 (PMC7793838; doi:10.3389/fonc.2020.582396)
Supplement: Supplementary file 1 [file DataSheet_1.pdf]

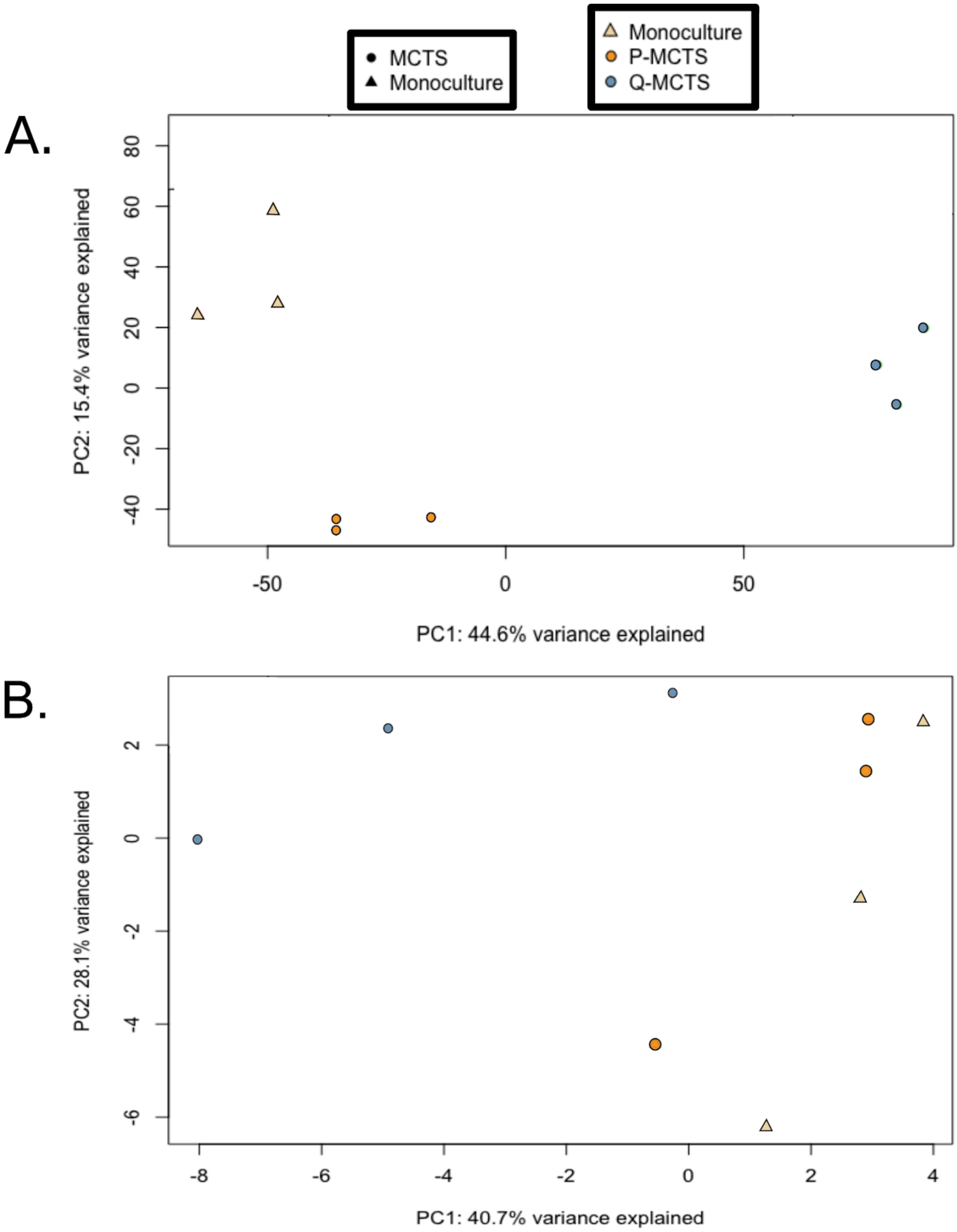

Figure S1: **Principal Component Analysis after data normalization. A**. Distribution of the samples after normalization for the mRNA expression data. **B**. Distribution of the samples after normalization for the miRNA expression data.
