# Supplementary material for: MicroRNAs Regulate Metabolic Phenotypes During Multicellular Tumor Spheroids Progression
Source: Front Oncol. 2020 Dec 4;10:582396. doi: 10.3389/fonc.2020.582396 (PMC7793838; doi:10.3389/fonc.2020.582396)
Supplement: Supplementary file 2 [file DataSheet_2.pdf]

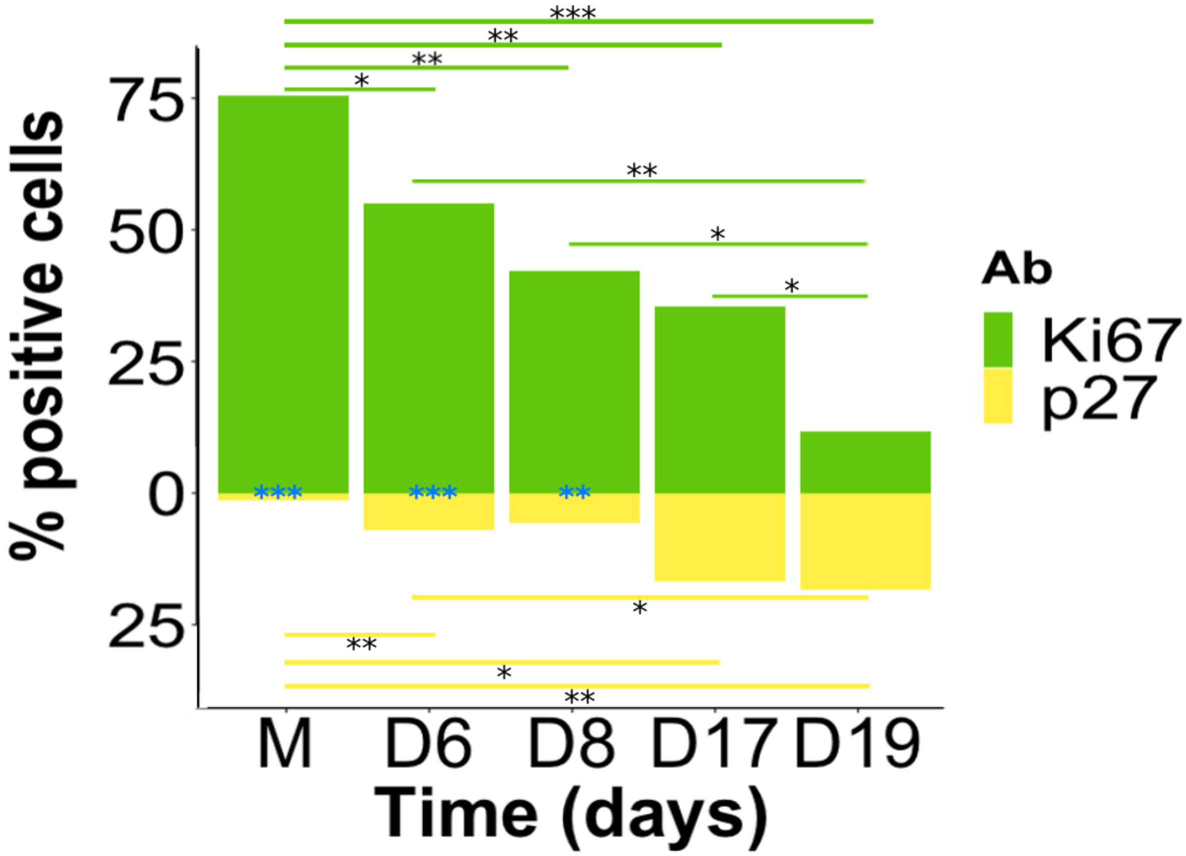

Figure S2: **Statistical Differences in Growing conditions**. Immunophenotyping of MCTS subpopulations in all temporal conditions: Monoculture (M) and MCTS day 6, 8, 17, and 19 in **raw** measurements. The statistical comparisons between temporal conditions for Ki67 and p27 are depicted in green lines and yellow lines, respectively. Additionally, black and blue asterisk represents the significance level in the comparison between temporal condition and within the temporal condition, respectively. The statistical differences are calculated using a t-test with equality of variance and the statistically significant results are shown below:

Ki67: M-D6: P= 0.015 (t= 4.0905; df= 4), M-D8: P= 0.0026 (t= 6.6971; df= 4), M-D17: P=0.0026 (t= 6.6937; df= 4), M-D19: P=0.000462 (t= 10.52; df= 4), D6-D19: P=0.00409 (t= 5.9169; df= 4), D8-D19: P= 0.014 (t= 4.1897; df= 4) and D17-D19: P= 0.042 (t= 2.9495; df= 4). **p27**: M-D6: P= 0.00724 (t= -5.0491; df= 4)**,** M-D17: P=0.02 (t= -3.7705; df= 4), M-D19: P= 0.006 (t= -5.3701; df= 4) and D6-D19: P= 0.0368 (t= -3.0839; df= 4). **Both**: M: P= 3.60E-06 (t= 35.873; df= 4), D6: P= 5.248E-04 (t= 10.178; df= 4) and D8: P= 0.00263 (t= 6.6695; df= 4).
